# DNA Oligonucleotides as Antivirals and Vaccine Constituents against SARS Coronaviruses: A Prospective Tool for Immune System Tuning

**DOI:** 10.3390/ijms24021553

**Published:** 2023-01-13

**Authors:** Volodymyr V. Oberemok, Oksana A. Andreeva, Edie E. Alieva

**Affiliations:** Department of Molecular Genetics and Biotechnologies, Institute of Biochemical Technologies, Ecology and Pharmacy, V.I. Vernadsky Crimean Federal University, 295007 Simferopol, Crimea

**Keywords:** antisense oligonucleotides, antisense antivirals, oligonucleotide adjuvants, oligonucleotide vaccines, SARS coronaviruses, innate immunity, adaptive immunity

## Abstract

The SARS-CoV-2 pandemic has demonstrated the need to create highly effective antivirals and vaccines against various RNA viruses, including SARS coronaviruses. This paper provides a short review of innovative strategies in the development of antivirals and vaccines against SARS coronaviruses, with a focus on antisense antivirals, oligonucleotide adjuvants in vaccines, and oligonucleotide vaccines. Well-developed viral genomic databases create new opportunities for the development of innovative vaccines and antivirals using a post-genomic platform. The most effective vaccines against SARS coronaviruses are those able to form highly effective memory cells for both humoral and cellular immunity. The most effective antivirals need to efficiently stop viral replication without side effects. Oligonucleotide antivirals and vaccines can resist the rapidly changing genomic sequences of SARS coronaviruses using conserved regions of their genomes to generate a long-term immune response. Oligonucleotides have been used as excellent adjuvants for decades, and increasing data show that oligonucleotides could serve as antisense antivirals and antigens in vaccine formulations, becoming a prospective tool for immune system tuning.

## 1. Introduction

Cell life, similar to the life cycle of viruses, is controlled by nucleic acids. Nevertheless, it is undeniable that proteins play a leading role in the creation of vaccines and as targets for the action of antiviral drugs. DNA and RNA remain overshadowed by proteins in antiviral development, mostly used by researchers as a matrix for the creation of proteins. However, nucleic acids are shown to be immunogens, which allow them to be used in vaccine formulations, and antisense nucleic acid sequences can act as blockers of virus gene expression. All these qualities endow them with the ability to prevent and treat various diseases, including diseases caused by SARS coronaviruses.

## 2. Antisense Antivirals

The first antisense oligonucleotide used as an antiviral preparation was used against the Rous sarcoma virus in the 1970s by Paul Zamecnik and Mary Stephenson [1]. Antisense technologies continued to rapidly develop in the 2000s in the elaboration of therapy against the human immunodeficiency virus (HIV) by groups of scientists led by Xiaobin Lu [2] and Jens Kurreck [3,4], as well as for the hepatitis C virus (HCV), by groups of scientists led by Muriel Soler [5] and Takanori Yokota [6].

An interim success for the development of antisense antivirals was the drug fomiversen (Vitravene) produced by ISIS Pharmaceuticals in 1998, the first modified antisense oligonucleotide with a phosphorothioate (PS) backbone approved by the FDA for the treatment of cytomegalovirus retinitis in immunocompromised HIV patients [7]. Fomiversen was actively used for the first three years, and then was discontinued due to an inaccurate mechanism of action. In particular, the role of RNase H in the antisense effect, low degree of effect, etc., was disputed [8]. However, the most significant factor for drug withdrawal was due to commercial reasons rather than serious side effects. Despite its withdrawal, fomiversen had a major impact on medicinal sciences because it demonstrated the concept of gene silencing in medicine [9]. Currently, there are 11 approved drugs for suppressing gene expression, mainly based on antisense oligonucleotides (9 drugs), 5 of which are with PS modification [10], including anti-SARS-CoV-2 preparations [11].

In theory, use of antisense DNA oligonucleotides during gene expression can disrupt transcription, splicing, and translation. They employ four main mechanisms of action in eukaryotic cells. The first mechanism is steric blocking of protein synthesis. The second mechanism is based on the action of RNase H, which mediates the degradation of the mRNA–antisense oligonucleotide complex. The third mechanism of action relies on the ability of antisense oligonucleotides to form a triple helix by binding to DNA strands, which leads to inhibition of transcription initiation. The fourth mechanism is associated with the formation of the antisense oligonucleotide–mRNA complex, which can affect splicing [12]. Due to the flexibility of the approach, antisense oligonucleotides can be used against different SARS coronaviruses that are always ‘sweeping their tracks’ during microevolution [13,14].

Coronaviruses are spherical, enveloped, positive-sense single-stranded RNA viruses [15]. They belong to the sub-family Orthocoronavirinae, family Coronaviridae, and order Nidovirales. Four of the human coronaviruses (HCoV-229E, HCoV-NL63, HCoV-HKU1, and HCoV-OC43) are not dangerous, and cause mild infections such as the seasonal common cold. In contrast, three other human coronaviruses (i.e., SARS-CoV-1 [16], MERS-CoV [17], and SARS-CoV-2 [18]) have been implicated in severe respiratory and multi-organ disorders [19]. SARS-CoV-1 and SARS-CoV-2 are both from the subgenus Sarbecovirus (SARS1, with a lethality of around 10%), whereas MERS-CoV is from subgenus Merbecovirus, with a lethality of around 30%. Both sarbecoviruses and merbecoviruses belong to the Beta genus of family Coronaviridae [20].

Shi et al. (2005) were among the first scientists to develop and implement antisense oligonucleotides as antivirals against SARS coronaviruses. They found some good target sites for antisense downregulation of SARS-CoV gene expression. Their results indicated a sequence-specific downregulation effect of antisense PS oligonucleotides (20mer) in Vero E6 cells, and the authors found an effective range of concentrations in which the antisense oligonucleotides inhibited expression of the E, M, and N genes of SARS-CoV in a concentration-dependent manner [21].

A radically new approach uses computer modelling, taking into account the avalanche of accumulated databases of SARS-CoV-2 genomic sequences from the current pandemic [22,23,24]. Sun et al. (2021) designed antisense oligonucleotides using in silico tools to disrupt the interactions between SARS-CoV-2 5′-RNAs and host proteins needed to regulate the viral infection cycle [25].

Hasan et al. (2021) conducted in vitro experiments on the human cell line Huh7.5.1 and reported a ~50% decrease in SARS-CoV-2 RNA yield in treated cells. They designed antisense oligonucleotides that targeted structurally conserved regions of SARS-CoV-2 RNA (ORF1 and N) that reduced the viral infection ratio by 50% in the human cell line Caco-2, which was derived from human colon carcinoma cells. Cells were transfected with antisense oligonucleotides using lipofectamine; the transfection mechanism involved the formation of cationic liposomes that enabled the internalization of anionic antisense oligonucleotides. Then, when in contact with the cell membrane, the liposomes created endosomes that entered the cell [26]. An innovative in silico tool proposed for antisense oligonucleotide design succeeded in targeting different regions of the rapidly evolving viral genome and reduced viral yield and infection [27]. SARS-CoV-2 and coronaviruses are known to rapidly evolve through point mutations as well as recombination, and the appearance of heavily mutated receptor-binding domains (RBD) through successive point mutations is the most parsimonious scenario [28,29,30]. The emergence of the Omicron VoC was a great example, because it accumulated an unusually high number of mutations at the spike, which had not been observed before in any other VoC, and led to immune escape [31]. In this regard, use of conservative antisense oligonucleotides is a prospective tool for immune system tuning.

The use of new approaches in biochemistry has helped to create modified antisense oligonucleotides with complex structures. Su et al. (2021) reported on the design and construction of chimeric oligonucleotides comprising a 2′-OMe-modified antisense oligonucleotide and 5′-phosphorylated 2′-5′ poly(A)4 (4A2-5) to degrade envelope and spike RNAs of SARS-CoV-2 [32]. The oligonucleotide was used for searching and recognizing target viral RNA sequences, and the conjugated 4A2-5 was used for guided RNase L activation to target well-conserved regions of the genome and degrade viral RNAs. As single-stranded RNA can be cleaved by RNase L during the innate antiviral response, degradation efficiencies with these chimeras were twice as potent as those with only antisense oligonucleotides for both SARS-CoV-2 RNA targets.

Another promising therapeutic strategy is by circular RNAs (circRNAs), a type of noncoding RNA that is present in all eukaryotes. Back-splicing of certain pre-mRNA exons give origin to circRNAs. Pfafenrot et al. (2021) systematically tested a series of antisense-circRNAs targeting the SARS-CoV-2 genome RNA, specifically its structurally conserved 5′-untranslated region. Functional assays revealed that specific segments of the SARS-CoV-2 5′-untranslated region could be efficiently accessed by specific antisense-circRNAs, resulting in a reduction in virus proliferation of up to 90% in cell culture, with a durability of at least 48 h. The authors showed that the activity of antisense-circRNA was surprisingly robust against point mutations in the target sequence [33].

Using an appropriate delivery system is a critical aspect for effective clinical applications of antisense oligonucleotides in both therapeutic and prophylactic contexts. The delivery of naked or unprotected antisense sequences could potentially expose them to enzymatic degradation, resulting in several negative side effects including toxicity, instability, and reticuloendothelial system and kidney clearance. Therefore, it is essential to optimize specific nanocarriers for the delivery of these fragile molecules to their target site [34]. Various types of nanoparticles including organic (i.e., lipid, polymer, and dendrimer), inorganic (i.e., gold), and virus-like or self-assembling protein nanoparticles were investigated against viral infections, including SARS or MERS coronaviruses. In each case, the physicochemical properties of nanoparticles such as the shape, size, and surface charge could considerably influence the success of the treatment. Though antisense oligonucleotides can be designed to detect and treat SARS-CoV-2 infection, the delivery of antisense oligonucleotides to lung tissue remains a great challenge [34,35,36].

Although there is no specific antiviral treatment recommended for MERS-CoV, antisense oligonucleotides can downregulate gene expression in MERS-CoV in a manner similar to SARS-CoV and SARS-CoV-2 to reduce MERS-CoV replication in treated cells.

Generally, antisense oligonucleotides are well-tailored to the fast microevolution of SARS coronaviruses and their genomes in databases may help create very conservative sequences with relevant nucleotide modifications to provide a long operational half-life [37].

## 3. Oligonucleotide Adjuvants

The last two decades have witnessed a revolution in our understanding of how the innate immune system captures micropathogens, which has provided huge opportunities to gain an understanding of the design and development of adjuvants [38], or ‘dirty little secrets’ (as posed by Charles Janeway) [39], to trigger the adaptive immune system through conserved components of micropathogens, known as pathogen-associated molecular patterns. Promising adjuvants include phosphorothioate oligonucleotides containing unmethylated CpG motifs, which are a distinctive feature of the genomes of bacteria and viruses.

One of the first studies on the use of CpG oligonucleotides was by Anna Bielinski et al. (1990), who investigated the regulation of gene expression using double-stranded phosphorothioate oligonucleotides on the human immunodeficiency virus. Modified oligonucleotides accumulated in cells more efficiently than unmodified double-stranded oligonucleotides and modulated gene expression in a specific way [40].

A significant contribution to the use of CpG oligonucleotides was also made by Arthur Krieg et al. (1995). They showed that phosphorothioate CpG motifs caused more than 95% of all spleen B cells to enter the cell cycle. The obtained data suggested a possible evolutionary link between the immune defense based on microbial DNA recognition and the phenomenon of ‘CpG suppression’ in vertebrates [41].

In vivo studies on mice with gene knockout and in vitro studies with a cell-based analysis of TLR9 (toll-like receptor 9) activation found that TLR9 is a cellular receptor, or pattern recognition receptor, for CpG oligonucleotides [42,43,44]. In mammals, TLR9 is mainly expressed in dendritic cells, monocytes/macrophages, and B cells [45,46,47]. Activation of TLR9 by CpG oligonucleotides leads to several immunological effects, including activation of dendritic cells, monocytes, macrophages, and NK cells, leading to the presentation of antigen and cytokine production. In addition, TLR9 induction activates B cells and increases B cell proliferation.

Synthetic oligonucleotides are powerful immunostimulants that have been studied for their use in the treatment of tumors, allergies, and infectious diseases, and as a vaccine adjuvant in humans. The immunostimulating effects of CpG oligonucleotides as vaccine adjuvants and their antimicrobial functions in domestic animals and bony fish are also being investigated [48].

Hui-Tsu Lin et al. (2021) confirmed that rS1-adjuvanted with fucoidan/trimethylchitosan nanoparticles are good nanocarriers and adjuvant candidates for intramuscularly administered CpG oligonucleotide-adjuvanted SARS-CoV-2 rS1 protein vaccine. In the murine model, the rS1/CpG/NP formulation increased the longevity and breadth of NT activity; Th1-biased responses were induced by the formulation of a broad-spectrum IgG response. According to the results of the study, the authors suggested that the rS1/CpG/NP formulation was a promising COVID-19 vaccine candidate [49]. Currently, according to a coronavirus vaccine tracker (12 December 2022), there are 50 approved SARS-CoV-2 vaccines (mRNA, inactivated, ChAdOx1, protein, Ad 26, and Ad 26- Ad5) [50].

Nanishi et al. (2022) found that an aluminum hydroxide and CpG adjuvant formulation (AH-CpG) produced an 80-fold increase in the anti-receptor binding domain, neutralizing antibody titers in both age groups compared with aluminum hydroxide alone and protecting aged mice from a SARS-CoV-2 challenge. The AH-CpG elicited neutralizing antibodies against both wild-type SARS-CoV-2 and the B.1.351 (Beta) variant at serum concentrations comparable to those induced by the licensed Pfizer-BioNTech BNT162b2 mRNA vaccine. The AH-CpG formulation induced similar cytokine and chemokine gene enrichment patterns in the draining lymph nodes of both young adult and aged mice and enhanced cytokine and chemokine production in human mononuclear cells of younger and older adults [51].

Yuntao Zhang et al. (2022) found that both novel low-dose and high-dose adjuvanted inactivated SARS-CoV-2 vaccines with CpG and Alum induced high levels of specific IgG antibodies and neutralizing antibodies against SARS-CoV-2, and the continuous stability of high-level neutralizing antibody titers over time showed that it had good, long-lasting immunity properties. Moreover, the vaccine exhibited equal effectiveness against the Beta, Delta, and Omicron variants through two immunization doses in rats, demonstrating its immune spectral properties. Positive correlations have been shown between virus-specific IgG antibody titers and COVID-19 severity [52].

Thus, CpG oligonucleotides are strong adjuvants making connections between innate and adaptive immune systems. Chemical synthesis of oligonucleotides [53] is currently widely used in molecular biology and medicine to create primers and probes for the diagnosis of diseases [54], gene assembly [55], vector inserts [56], genome sequencing [57], genomic editing [58], and gene modification [59], as well as the development of drugs using antisense therapy [60,61]. The method most widely used in the synthesis of oligonucleotides is the automatic solid-phase phosphoramidite synthesis method, which makes it possible to obtain the specified sequences of oligonucleotides relatively quickly, with high yield and purity [62], for use in solving a wide range of problems.

For the human population, COVID-19 is the third significant coronavirus infection to occur in the twenty-first century, following severe acute respiratory syndrome (SARS) in 2002–2003 and Middle East respiratory syndrome (MERS) in 2012. MERS is still ongoing, with a total of 2601 laboratory-confirmed cases of Middle East respiratory syndrome (MERS) reported globally and 935 associated deaths at a case-fatality ratio (CFR) of 36%, from April 2012 to November 2022. The majority of these cases were reported in Saudi Arabia, with 2194 cases and 854 related deaths (CFR: 39%) [63]. While those outbreaks alerted the world to the virulent potential of the coronavirus family, SARS fizzled out before a vaccine could make its way through clinical trials, and MERS was not viewed seriously enough to generate sustained funding for vaccine developers [64,65]. CpG oligonucleotides in possible vaccine formulations against SARS-CoV and MERS-CoV would be also effective.

## 4. Oligonucleotide Vaccines

Scientists have collected sporadic but convincing data over the past decades on the possibility of using nucleic acids as an active immunogen [66,67,68,69,70,71].

The sequence of bacterial DNA, which has the unmethylated cytosine-guanine dinucleotide CG at its core (CpG), activates the mammalian immune system to produce antibodies rather than the DNA backbone [67]. As this unusual protein DNA enters the host in a similar way to foreign DNA, there is an antigenic reaction, as its epitope structure carries sequences not shared with the host. Data have demonstrated that this reaction to foreign DNA is universal and independent of immunity status; some studies have shown an anti-DNA reaction in hosts with normal or aberrant immune systems. As described by David Pisetsky in 1998, with the recognition of the epitope structure and immunostimulatory properties of bacterial DNA, DNA has been transformed from a uniformly inert substance into one that is powerful and pervasive [72]. Until 2020 [64], although oligonucleotides themselves had been employed in vaccine formulations as adjuvants for many years, not a single attempt was undertaken to build a functioning vaccine completely based on these structures [73,74].

The current generations of coronavirus vaccines all use entire proteins or protein fragments to stimulate an immune response. The idea of oligonucleotide vaccines was first proposed by Oberemok et al. [64] in the form of a lasso-like oligonucleotide phosphorothioate (PS) construct containing an antigen-presenting ‘head’ with a unique sequence for activating acquired immunity, a tail with CpG islands to activate innate immunity, and a ‘neck’ connecting the ‘head’ and ‘tail’. In the proposed ‘lasso’ (La-S-so, lamellar anti-SARS-CoV-2 sulfur-containing oligonucleotide) construction, the CpG motifs were placed in the ‘tail‘ region in the most efficient 5′-purine-purine-unmethylated deoxycytosine-deoxyguanosine-pyrimidine-pyrimidine -3′ position [64].

The capacity of “La-S-so” oligonucleotide vaccines to activate adaptive immunity and function as efficient immunogens capable of inducing a potent immune response are key to their efficacy. One of the most important questions is how the viral particle inside a virion is neutralized by antibodies made on the nucleic acid. Antibodies produced in response to the La-S-so vaccine will not be able to attack the mature viral particle, as the virion contains the nucleic acid inside. We assume that the human body contains antibodies that can enter human cells during viral infection and target particular fragments of nucleic acids in RNA viruses. Generally speaking, antibodies do not easily pass through intact cellular or subcellular membranes in living cells [75]; however, this is not always the case. Numerous investigations conducted in cultured cells over the years have demonstrated that it is possible to facilitate the cellular internalization of antibodies [76]. The potential for in vivo therapeutic advantages of a nuclear-penetrating lupus anti-DNA autoantibody has also been shown in several studies [77,78].

Another important question is whether dendritic cells are capable of presenting vaccine fragments containing the unique nucleic acid sequence of viruses. Dendritic cells are leukocytes that are obtained from bone marrow, and are another key to a strong immune response. These cells can migrate throughout the body, during which they are sparsely distributed. The specialization of these cells is in the uptake, transport, processing, and presentation of antigens to T cells [79,80]. In order to present antigens to T lymphocytes, dendritic cells must move to the secondary lymphoid organs. At the same time, the subsequent presentation of antigens to T lymphocytes initiates an immune response that is antigen-specific. While there are other antigen-presenting cells, the processes that make dendritic cells particularly effective at stimulating the immune response are different, with meticulous regulation of each step (antigen uptake, intracellular transport and degradation, and loading of MHC). So far, not a single antigen has been found that actively engages only B cells without affecting T cells, the versatility of whose activity depends on dendritic cells [81]. At the same time, it is known that nucleic acids affect B cells and this interconnection between B and T cells will likely be demonstrated in the near future.

In a number of our experiments with SARS-CoV-2, a semi-natural vaccine of the La-S-so type was found to have a moderate immune response, expressed by formation of specific antibodies. In addition, studies of the La-S-so vaccine were conducted in humanized mice with the human ACE2 receptor. By day 30 of the experiment, it was shown that while all the vaccinated animals were alive and beginning to recover, all the control mice had died. The morphological parameters of the lung parenchyma in vaccinated animals were comparable to those of intact animals (under publication). We assume that these observed effects cannot be solely explained by the adjuvant role of oligonucleotides. Research has continued on the selection of doses (fine tuning to discover the most effective amount) and refinement of constructs such as ‘La-S-so’ to find those with the best immune response [82].

**Figure 1:**
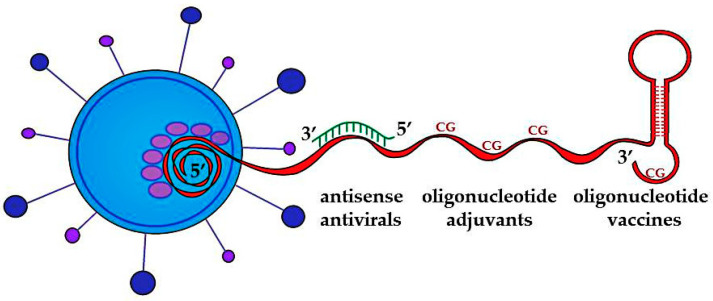
Genetic material of SARS coronaviruses as a source for creation of antisense antivirals, oligonucleotide adjuvants, and oligonucleotide vaccines.

Thus, studies have shown that oligonucleotide constructs have great potential in the fight against SARS coronaviruses. The genetic material of SARS coronaviruses can be a source for the creation of antisense antivirals, oligonucleotide adjuvants, and oligonucleotide vaccines (Figure 1), and even the most daring ideas can come true in the postgenomic era of biology development.

## 5. Conclusions

The greatest advantage of the use of nucleic acids in practical applications lies in the fact that they are universal molecules and effective managers of the functional processes of the cell. The discovery in the field of the practical uses of nucleic acids as active tools of influence on cells has revolutionized biology. While there are no available low-cost antiviral drugs based on nucleic acids yet, it is only a matter of time before such drugs are developed. This is a promising approach as nucleic acids are natural biopolymers with predictable safety and efficacy. There is also the possibility of creating targeted drugs not only for specific diseases, but also for specific people. Oligonucleotide vaccines and antiviral drugs based on oligonucleotides could create a new spiral in the evolution of drugs for the prevention and treatment of viral diseases, including those caused by SARS coronaviruses.
